# Vitamin D Deficiency is Associated with Increased Use of Antimicrobials among Preschool Girls in Ethiopia

**DOI:** 10.3390/nu11030575

**Published:** 2019-03-07

**Authors:** Johanna Bodin, Adane Mihret, Carol Holm-Hansen, Jennifer L. Dembinski, Mai-Chi Trieu, Bamlak Tessema, Azeb Tarekegne, Solomon A. Yimer, Rebecca Cox, Abraham Aseffa, Bjørn Haneberg, Siri Mjaaland

**Affiliations:** 1 Department of Infectious Disease Immunology, Norwegian Institute of Public Health, 0456 Oslo, Norway; johanna.bodin@fhi.no (J.B.); carol.holm-hansen@fhi.no (C.H.-H.); jenniferlynn.dembinski@fhi.no (J.L.D.); bjha1@hotmail.com (B.H.); 2 K.G. Jebsen Centre for Influenza Vaccine Research, University of Oslo, 0316 Oslo, Norway; chi.trieu@uib.no (M.-C.T.); rebecca.cox@uib.no (R.C.); 3 Armauer Hansen Research Institute, 1005 Addis Ababa, Ethiopia; amihret@gmail.com (A.M.); bamlak.tessema@yahoo.com (B.T.); azititar@gmail.com (A.T.); aseffaa@gmail.com (A.A.); 4 Department of Virology, Norwegian Institute of Public Health, 0456 Oslo, Norway; 5 The Influenza Centre and Department of Clinical Science, University of Bergen, 5020 Bergen, Norway; 6 Coalition for Epidemic Preparedness Innovations (CEPI), 0306 Oslo, Norway; solomon.yimer@cepi.net; 7 Department of Microbiology, University of Oslo, 0316 Oslo, Norway

**Keywords:** influenza, respiratory viral and bacterial infections, vitamin D, sex/gender, nutrition

## Abstract

Preschool children in Addis Ababa, Ethiopia, are highly exposed to influenza viruses. Factors related to infections, nutrition, and environmental conditions that might explain the burden of influenza among these children were investigated. Ninety-five preschool children, 48 girls and 47 boys, were followed clinically for 12 months. Illness and immune responses to influenza; three other respiratory viruses; five airway pathogenic bacteria; and levels of vitamins D, A, and B12 were assessed. Most of the children had antibodies to numerous respiratory viral and bacterial agents at study start, and many were infected during follow-up. Twenty-five girls and 25 boys fell ill during the study, and were treated with one or more courses of systemic antimicrobials. Ninety percent of both girls and boys had 25-hydroxyvitamin D [25(OH)D] levels below the recommended levels. While there was no overall difference in the levels of vitamins D, A, and B12 between girls and boys, treated girls had significantly lower 25(OH)D levels than non-treated girls and treated boys. There was a considerable number of short for age children, but only the short treated girls had significantly lower 25(OH)D levels than the non-treated children. Preschool girls with low 25(OH)D levels were more vulnerable to pathogenic microbes than boys.

## 1. Introduction

We have recently found that preschool children living in Addis Ababa are heavily exposed to several strains of influenza virus [1]. Influenza [2,3] and infections due to parainfluenza virus, respiratory syncytial virus, metapneumovirus, and adenovirus may predispose for bacterial pneumonia [4,5,6], which is a leading cause of death among children below the age of five [7]. Recent studies addressing influenza-associated disease documented a substantial burden among preschool children in Bangladesh and Kenya [8,9].

Despite remarkable reductions in infant and child mortality rates in Ethiopia over the last 25 years, mortality is still unacceptably high [10]. This reduction in mortality is partially a result of the introduction of new vaccines during the past few years. Results of numerous studies have documented the impact and effectiveness of pneumococcal vaccines on reducing hospitalization and mortality among children under five [11]. However, even the most effective vaccines against pneumococci may fail to protect immunized individuals [12].

More than twenty years ago, rickets was highly prevalent among children below the age of five admitted to hospital in Addis Ababa with pneumonia [13]. A recent study documented that a significant number of schoolchildren from Central Ethiopia, a country with “thirteen months of sunshine”, had lower than recommended levels of 25-hydroxy vitamin D [25(OH)D]. This deficiency was primarily found among children with less exposure to sunlight, and was more frequent among girls than boys [14].

Contrary to what was expected, vitamin D deficiency in schoolchildren from Central Ethiopia was associated with overweight or obesity [15]. Regardless, malnutrition cannot be ruled out as one of the factors contributing to vitamin D deficiency.

In the present study, 95 of the 103 children who were included in our previous study [1] were followed for one year. In addition to clinical examinations and possible treatment, blood samples were obtained to determine immune status for exposure to several influenza strains, other common respiratory viruses, and selected airway pathogenic bacteria, as well as for the analysis of vitamins D, A, and B12 that are essential for a well-functioning immune system. Our hypothesis was that influenza affects morbidity (use of antimicrobials), and the aim was to investigate factors related to viral and bacterial infections, nutrition, and environmental conditions that may explain the burden of influenza among preschool children in Addis Ababa, Ethiopia. The results indicate that recurring airway infections and vitamin D deficiency are prevalent and as frequent among preschool girls as boys in Ethiopia. Illness, defined as the use of antimicrobials, and increased risk of multiple respiratory infections are associated with low 25(OH)D levels in girls but not in boys.

## 2. Methods

### 2.1. Study Population

This study is a follow-up of a cross-sectional cohort study designed to investigate the prevalence of influenza and its effect on child health in a sub-tropical climate, Addis Ababa, Ethiopia (9°0′49.75″ N/38°42′21.49″ E). Ethiopian preschool children were invited to participate in a 12-month follow-up surveillance of health status from March 2014. Any new infection, the need for antimicrobial treatment, and social status were investigated. As previously described [1], 103 children (54 girls/49 boys, about one fourth of all children in a kebele/community), aged 2–5 years (23–60 months), were selected using a systematic sampling procedure from all the healthy children residing in Woreda 01, Kolfe Kerano sub city of Addis Ababa. Exclusion criteria were human immunodeficiency virus (HIV) infection and chronic illness. Blood samples with a sufficient volume for cell isolation were collected from 95 children (48 girls/47 boys) at the start of the study. More than 80% of girls and boys had received BCG, oral polio-vaccine, and vaccines against diphtheria, tetanus, pertussis, Hepatitis B, Hib-infection, and measles. Only three, two girls and one boy, had received a pneumococcal conjugate vaccine. The 95 children were followed over a 12-month period and clinical data was registered monthly. Seventy of the children (39 girls/31 boys) provided a blood sample at the end of the study period, shown in Table 1.

Written informed consent was obtained from the parents or next of kin for all participants. The study was approved by the Regional Committees for Medical Research Ethics—South East Norway (reference number 2012/2183), AHRI/ALERT (reference number PO32/13), and the National Health Research Ethics Review Committee of Ethiopia (reference number 3.10/447/06), and procedures according to the Helsinki Declaration (1964, 2008) were followed.

### 2.2. Clinical Examination and Socio-Economic Status

A clinical examination was performed by a medical doctor at the start and end of the study (*n* = 95 children). Trained Health Extension Workers (HEW) performed monthly clinical examinations. A medical doctor was contacted to provide additional examination and possible antimicrobial prescription when a child was ill. We refer to the children who received systemic antimicrobial treatment as “sick” and the non-treated children having mild or no clinical symptoms as “well” in our analyses. Height and weight were measured at the start of the study. Socio-economic information, including parental level of education, occupation, monthly income, living conditions, exposure to indoor cigarette smoke or cooking smoke, and breastfeeding, representing factors important for the spread of, and predisposition to, respiratory tract infections, were recorded at the start.

### 2.3. Sample Collection

Venous blood from 95 children was collected at the start of the study in acid citrate dextrose tubes (BD Vacutainer; Becton, Dickinson and Company, Franklin Lakes, NJ, USA), as previously described [1]. Blood samples were diluted 1:1 in 0.9% NaCl, and plasma and peripheral blood mononuclear cells (PBMC) were separated by Ficoll-Paque density gradient centrifugation (Ficoll-Paque Premium 1.077; GE Healthcare, Chicago, IL, USA) using SepMate 50 mL tubes (STEMCELL Technologies UK Ltd., Cambridge, UK) following the manufacturer’s instructions.

A second blood sample was obtained from 70 children (39 girls, 31 boys) after 12 months (M12). Seventy paired plasma samples were obtained and sufficient numbers of PBMC were isolated from 63 paired samples (D0 and 12M) (Table 1). Cells were stored at −150 °C in 25% fetal calf serum/10% dimethyl sulfoxide (DMSO)/65% AIM-V cell culture medium (Gibco; Thermo Fisher Scientific, Waltham, MA, USA), and plasma was stored at −80°C prior to use.

### 2.4. Hemagglutination Inhibition Assay

The hemagglutination inhibition (HI) assay was performed to assess antibodies to influenza strains A/HINI, A/H3N2, and B, as described by Dembinski et al. [1]. The 70 paired plasma samples were analyzed in duplicate as previously described [16]. In short, plasma samples were treated with a receptor-destroying enzyme (RDE), a lyophilized culture supernatant of Vibrio cholerae Ogawa type 558 (Denka Seiken Co., Ltd., Tokyo, Japan). The samples were thereafter analyzed in duplicate using 0.7% turkey red blood cells with eight hemagglutinating units (HAU) of β-propiolacetone-inactivated influenza A and ether-extracted influenza B strains, as previously described [16]. The HI antibody titer was determined as the reciprocal of the highest plasma dilution, causing 50% inhibition of hemagglutination. Negative titers (≤10) were assigned a value of five for calculation purposes. Intermediate values represent geometric mean titers of repeated testing. A positive titer was defined as HI titer >10. A new infection during the 12-month follow-up was defined as at least a four-fold increase in HI titer in the 12-month sample compared to the titer at study start [17].

### 2.5. Streptococcus FP23 Enzyme-Linked Immunosorbent Assay (ELISA)

Antibody levels against a non-encapsulated derivative of *Streptococcus pneumoniae*, the TIGR4 serotype 4 mutant FP23 (kindly provided by Francesco Iannelli, Siena, Italy), were determined by ELISA (70 paired plasma samples, D0 and 12M) using the method described by Kolberg et al. [18]. A positive response was defined as two standard deviations above zero in the standard curve. Inter assay precision was 5–10% deviation.

### 2.6. Ex Vivo Interferon Gamma (IFNγ) Enzyme-Linked ImmunoSpot (ELISpot) Assay

Influenza A/H1N1, A/H3N2, and *Streptococcus pneumonia*-specific IFNγ positive cell-mediated immune (CMI) responses were measured by an ex vivo ELISpot assay, according to the manufacturer’s instructions (CTL Europe GmbH, Bonn, Germany) [19]. CMI responses to influenza B were not measured due to insufficient PBMC. Briefly, 200,000 PBMC per well was stimulated with (a) 75 hemagglutination units (HAU) of whole inactivated viruses A(H1N1)/California/7/2009pdm09 or A(H3N2)/Brisbane/10/2007; and (b) 50 µL of a heat-inactivated *Streptococcus pneumoniae* FP23 (OD 0.65). Concanavalin A (5 µg/mL) and 0.28% DMSO in AIM-V medium (Gibco; Thermo Fisher Scientific) were the positive and negative controls, respectively [20]. A positive response was defined as two standard deviations above the negative control. Inter assay precision was 5–10% deviation. New infections during the 12 month follow-up were defined as at least a two-fold increase in spot forming units (SFU)/10^6^ PBMC compared to samples at the start.

### 2.7. Pneumoslide IgG Antibody Detection of Selected Viruses and Bacteria

Immunofluorescent detection of antibodies against adenovirus; respiratory syncytial virus; parainfluenza 1, 2, and 3; *Legionella pneumophila* serogroup 1; *Mycobacteria pneumoniae*; *Coxiella burnetii*; and *Chlamydophila pneumoniae* was performed using the Pneumoslide IgG Kit (VirCell, Granada, Spain), according to the manufacturer’s instructions. A positive response was defined as a signal higher than the negative control. A new infection during the 12-month period was defined as a positive signal in the follow-up sample compared to a negative sample at the start.

### 2.8. Vitamin Analyses

Diluted plasma samples obtained after PBMC isolation from 95 children were subjected to an analysis of vitamins D [25(OH)D], A, and B12 at Vitas AS, Oslo, Norway. Levels of 25(OH)D and vitamin A (retinol) were assessed with HPLC-MS/MS (Agilent Technologies, Palo Alta, CA, USA) in 100 μL diluted plasma. The lower limit of quantification (LLOQ) was 5 nmol/L for 25(OH)D and 0.05 μmol/L for vitamin A, with standard deviation of 4.2% from the reference. Vitamin B12 was determined by ELISA (Monobind, Lake Forest, CA, USA) in 25 μL diluted plasma, LLOQ = 90 pmol/L, with standard deviation of 8% to the reference. These analyses were only performed on samples collected at the start of the study. To calculate the plasma dilution factor resulting from PBMC isolation, blood from three healthy donors was drawn into two tubes; (1) acid citrate dextrose tubes and subsequent dilution 1:1 in 0.9% NaCl prior to PBMC isolation in SepMate tubes; and (2) EDTA tubes for direct centrifugation and plasma collection without dilution. By comparing levels of vitamins in plasma obtained from the healthy blood donors using SepMate and EDTA tubes, the mean dilution factor was 3.53. Vitamin deficiency was defined as plasma levels below the following limits: 50 nmol/L for 25(OH)D [14]; 0.7 µmol/L for vitamin A [21]; and 150 pmol/L for vitamin B12 [21].

### 2.9. Statistics

Statistical analyses were performed by the Mann-Whitney non-parametric *U* test using GraphPad. Linear regression was used to assess any association between 25(OH)D and vitamin A, and between 25(OH)D and vitamin B12. Kruskal–Wallis one-way analysis of variance was performed on the three groups of children with (=1) or without (=0) multiple infections categorized after 25(OH)D levels (<37, 37–50, and >50 nmol/L) and on the number of antimicrobial courses used in these three groups of children. The Chi^2^ test was performed on differences in socio-economic status between girls and boys. Significant differences were given when *p* < 0.05.

## 3. Results

### 3.1. High Prevalence of Antimicrobial Use during Follow-Up

During the study period, 50 (25 girls/25 boys) of the 95 children fell ill and were prescribed one or more courses of systemic antimicrobial agents by a medical doctor. These children were categorized as “sick”. Forty-three children (22 girls/21 boys) received diagnoses, including the common cold, upper respiratory tract infections, tonsillitis, pharyngitis, sinusitis, and otitis media, and 14 of these children (7 girls/7 boys) were diagnosed with pneumonia, as summarized in Table 2. Twenty-eight of the 50 “sick” children had combinations of multiple infections during the study period, including airway infections, intestinal parasites, acute gastroenteritis, and/or urinary tract infections. Forty-five children (23 girls/22 boys) were categorized as “well”, i.e., did not receive any antibacterial treatment during the one-year follow-up. Thirty-four (17 girls/17 boys) “well” children did not seek medical attention, whereas 11 (6 girls/5 boys) received other treatments, including local dermal antifungal or antiviral treatment during the surveillance period.

### 3.2. High Prevalence of Influenza and Pneumococcal Exposure during Follow-Up

**Figure 1:**
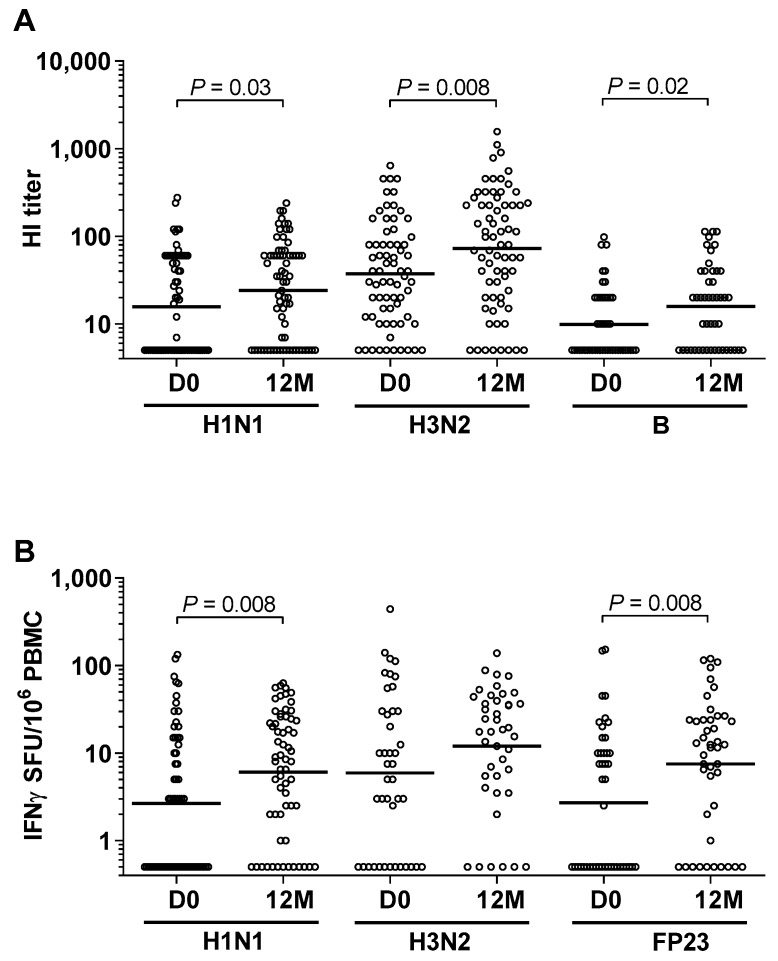
High prevalence of influenza and pneumococcal exposure during the one-year follow-up. (**A**) Humoral immune responses to influenza viruses were measured by hemagglutination inhibition (HI) antibody titers to influenza A/H1N1, A/H3N2, and B virus strains, in plasma from 70 preschool children at the study start (D0) and after 12 months’ (12M) observation; (**B**) Cellular immune responses to influenza viruses and *Streptococcus pneumoniae* were measured as spot-forming units SFU indicating interferon gamma IFNγ-positive cells per million peripheral blood mononuclear cells (PBMC) after stimulation with whole inactivated influenza A/H1N1, influenza A/H3N2, and FP23 from *Streptococcus pneumoniae*, respectively. Horizontal lines represent median values, and *p*-values were obtained by the Mann-Whitney *U*-test.

**Figure 2:**
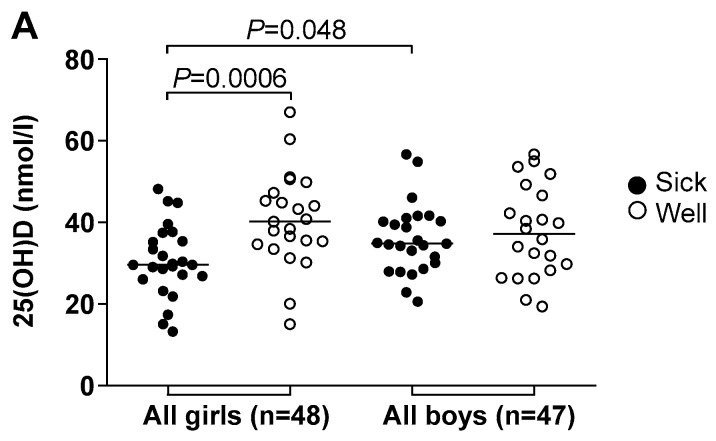

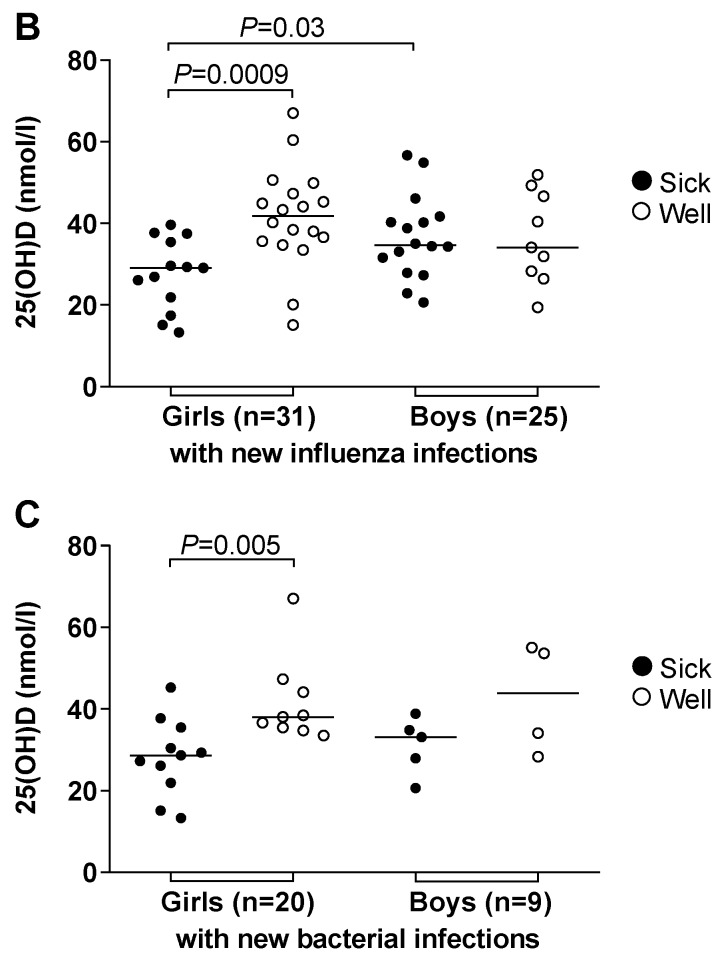
Vitamin D levels and illness, defined as the need for antimicrobial treatment. (**A**) 25(OH)D levels measured in plasma at the start of the study from 95 preschool children (girls and boys) treated with one or more courses of systemic antimicrobial agents (designated “sick”) or with no treatment (designated “well”); (**B**) Out of 70 children followed-up, 56 developed HI antibody titers or an increased cellular immune response to new strains of influenza virus; (**C**) 29 children developed antibodies to one or more of the four airway pathogenic bacteria, *Legionella pneumophila*, *Mycoplasma pneumoniae*, *Coxiella burnetii*, and *Chlamydophila pneumoniae*. Horizontal lines represent median values, and *p*-values obtained by the Mann-Whitney *U*-test were given if less than 0.05.

At day 0, there was a high prevalence of HI antibodies (96%) to influenza A/H1N1, A/H3N2, and B [1]. After 12 months, 56 (31 girls/25 boys) of the 70 children had been re-infected or infected with a new strain of influenza A/H1N1, A/H3N2, and/or B, as indicated by at least a four-fold increase in the HI antibody titer and/or a two-fold increase in IFN*γ*-positive SFU (Figure 1A,B and Figure 2B). The increase in the HI titer antibody was significant for all influenza A/H1N1, A/H3N2, and B strains (Figure 1A). The increase in CMI was significant for influenza A/H1N1, and the same tendency, although not significant, was seen for A/H3N2 (Figure 1B).

The mean antibody level to pneumococcal FP23 remained constant throughout the 12-month period (data not shown). In contrast, CMI against FP23 increased significantly during the year (Figure 1B). A two-fold or greater increase in IFNγ-positive SFU in 21 (from 13 girls/8 boys) of the 44 paired samples indicated new infections with *Streptococcus pneumoniae* during the one-year follow-up. Half of these children (6 of 13 girls/4 of 8 boys) received systemic antimicrobial treatment.

### 3.3. High Exposure to Other Airway Pathogenic Viruses and Bacteria

Almost all of the 70 children (39 girls/31 boys) who provided blood after 12 months had antibodies against parainfluenza 1, 2, 3, adeno- and/or respiratory syncytial viruses at study start (Table 3), and 85% of the children had been infected with all three viruses. The respiratory viral exposures were equally distributed among children categorized as “sick” or “well”. The gender distribution was also similar. Many children also developed antibodies to several new respiratory pathogens during the study, indicating multiple new infections (Table 3).

Of the 70 children, 29 (20 girls/9 boys) developed antibodies to one or more of the bacterial strains *Legionella pneumophila*, *Mycoplasma pneumoniae*, *Coxiella burnetii*, and *Chlamydophila pneumoniae* during the one-year follow-up. The positive responses were equally distributed among “sick” and “well” children (Figure 2C). These bacterial infections occurred more often among girls than boys (20 girls/9 boys), and half of the girls and boys became ill (Figure 2C). Multiple bacterial infections occurred more often among girls than boys (6 of 20 girls/1 of 9 boys) (Table 3). Influenza together with multiple bacterial infections was also more frequent among girls than among boys (24 of 39 girls/14 of 31 boys).

### 3.4. Vitamin D Deficiency Correlated to Illness and Antimicrobial Treatment

Almost 90% of the study population (*n* = 95) had 25(OH)D levels below the recommended 50 nmol/l [14] at the start of the study (Table 4). 25(OH)D levels were lower than the recommended level in 87% of the 70 children followed for one year. There was no sex or age difference associated with overall 25(OH)D levels (Table 1). Girls receiving systemic antimicrobial treatment (referred to as “sick”) during the study period had significantly lower 25(OH)D levels compared to “sick” boys (Figure 2A). The difference between 25(OH)D levels among “sick” and “well” girls was even more significant. The “sick” boys did not have significantly lower 25(OH)D levels than the “well” boys (Figure 2A).

Fifty-six children were infected with influenza during the study period. These infections were equally distributed among “sick” and “well” children, while 25(OH)D levels were significantly lower among the “sick” girls as compared to the “well” girls. The “sick” girls also had significantly lower 25(OH)D levels than the “sick” boys (Figure 2B).

Twenty-nine children developed antibodies to the four airway pathogenic bacteria *Legionella pneumophila*, *Mycoplasma pneumoniae, Coxiella burnetii*, and *Chlamydophila pneumoniae*. As observed with the new influenza infections, the “sick” girls had lower 25(OH)D levels than the “well” girls. The same trend was observed between the “sick” and “well” boys, but the difference was not significant (Figure 2C). 25(OH)D levels among 21 (13 girls/8 boys) of the 44 children infected with *Streptococcus pneumoniae* were also lower among the “sick” than “well”, although the difference was not significant.

The children were analyzed by groups with 25(OH)D levels of <37, 37–50, and >50 nmol/L [22]. There were significantly more girls with multiple infections in the lowest range of 25(OH)D compared to 25(OH)D levels above 50 nmol/L (Kruskal-Wallis one-way analysis of variance on ranks, *p* = 0.04, Supplementary Materials Figure S1). A similar correlation was not found among boys (Figure S1A). A significant increase in the use of antimicrobials, defined as three to six treatments compared to one to two treatments, was observed among girls within the lowest 25(OH)D category (Kruskal-Wallis one-way analysis of variance *p* = 0.03, Figure S1B).

### 3.5. Body Height and Weight

**Figure 3:**
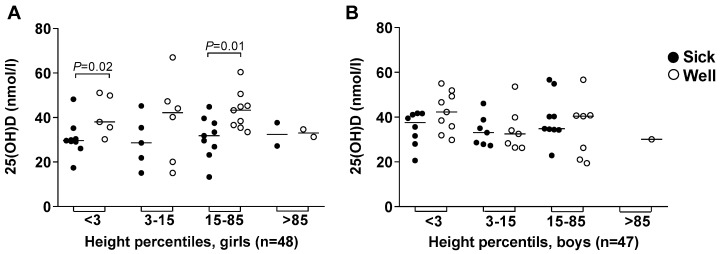

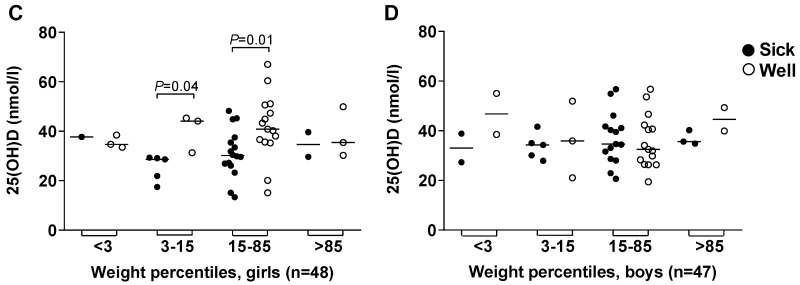
Vitamin D-levels related to body height and weight. (**A**) 25(OH)D levels measured in plasma at the start of the study from 48 preschool girls; and (**B**) from 47 preschool boys as related to height percentiles; and (**C**) from the same girls and (**D**) boys as related to weight percentiles, and defined on follow-up as “sick” or “well”. Horizontal lines represent median values, and *p*-values obtained by the Mann-Whitney *U*-test were given if less than 0.05.

Among the 95 children included at study start, 31 (13 girls/18 boys) were short for age (under third percentile), whereas only eight (4 girls/4 boys) children were underweight (Figure 3). Low 25(OH)D levels were associated with morbidity among the short girls, but not among the short boys (Figure 3A,B). There was also a significant association between 25(OH)D levels among “sick” as compared to “well” girls within the normal height (Figure 3A) and weight (Figure 3C) percentiles. None of these trends were observed among the boys (Figure 3B,D).

### 3.6. Vitamins A and B12

At study start, 70 of the children (*n* = 95) also had lower than recommended vitamin A levels (<0.7 µmol/L) [21] (Table 4). Fifty-one of the 70 children who were followed for one year had below the recommended level for vitamin A. No children were below the recommended level for vitamin B12 (<150 pmol/L) [21]. There was no sex or age difference associated with overall vitamin A or B12 levels (Table 1). There was no correlation between illness, i.e., antimicrobial treatment, and vitamin A levels, even when girls were compared to boys. There was an apparent correlated trend between vitamin D, A, and B12 levels, although not significant for vitamin A. The correlation between vitamin D and vitamin A was investigated with linear regression analysis (*n* = 95, r = 0.2, *p* = 0.076), as well as for vitamin D and vitamin B12 (*n* = 93, r = 0.3, *p* = 0.003).

### 3.7. Socio-Economic Status

Using questionnaire data (results not shown), there was no background variable that had a statistically significant gendered profile. No differences were found in socio-economic factors, including living conditions (number of people sleeping in the same room), or history of family indoor smoking or exposure to indoor smoky cooking, which are factors important for the spread of, and predisposition to, respiratory tract infections between “sick” and “well” children, and between girls and boys.

## 4. Discussion

The results of our former investigation showed that preschool children in Addis Ababa, Ethiopia, had been heavily exposed to multiple strains of influenza viruses [1]. During the follow-up, antibody responses indicated that many children had been re-infected with one or more of the same influenza strains, or infected with one or more of the strains to which they had not previously been exposed. Cellular immune responses induced by influenza A-viruses confirm that the children were more or less continuously exposed to influenza viruses.

High levels of antibodies to the pneumococcal FP23 protein were observed both at study start and end. This may be explained by a high incidence of *Streptococcus pneumoniae* carriage reported to be approximately 60% among children below five years in Sub-Saharan Africa [23]. T cell responses to FP23 were examined after 12 months and confirmed a significant increase in cellular immunity [24,25]. As previously reported [3], our results suggest that influenza might actually predispose for, or be associated with, pneumococcal infection among children in Ethiopia.

Using a rapid antibody test, Pneumoslide IgG, we found that almost all children had been infected with parainfluenza viruses, adenovirus, and respiratory syncytial virus, i.e., viruses associated with secondary bacterial infections, before study start [4,5,6]. With the exception of two children, those not infected at study start were infected during follow-up. Even though the techniques used were different, the high prevalence of these viral infections among children in Addis Ababa is in contrast to the moderate or low prevalence among children attending day care in Norway [26].

Pneumoslide IgG also revealed that a number of children had antibodies at the start to *Legionella pneumophila*, *Mycoplasma pneumoniae*, *Coxiella burnetii*, and *Chlamydophila pneumoniae*. Among 29 children who were not infected at the start, 37 new infections to one or more of these four pathogens were detected during follow-up, and girls were then more prone to be infected than boys. This observation is surprising considering that the prevalence and severity of both viral and bacterial infections are generally higher in boys than in girls [27].

Equal numbers of girls and boys appeared sick and were treated with systemic antimicrobial agents (categorized as “sick”) during follow-up. Whereas 25(OH)D levels were not significantly different between girls and boys, “sick” girls had lower 25(OH) D levels than “sick” boys. The “sick” girls, moreover, had much lower 25(OH)D levels than non-treated (“well”) girls. The same tendency was observed among the children who were infected with an influenza strain, either as a primary infection or reinfection. According to a recent systematic review, a higher risk of all-cause mortality was found to be related to vitamin D deficiency, and approximately half of all children admitted to intensive care units had lower than recommended vitamin D levels [28].

Multiple infections were significantly more frequent among girls with the lowest vitamin D levels. This is in accordance with reports of increased risk for hospitalization due to respiratory tract infections among adults with 25(OH)D levels below 37 nmol/L [22,29]. Furthermore, intervention studies addressing vitamin D supplementation show decreased respiratory infections at serum levels above 50 nmol/L [30,31], as well as the prevention of seasonal influenza among school children in Japan [32,33]. The girls in our study with the lowest 25(OH)D levels received the highest number of antimicrobial courses.

An unexpectedly high proportion, 27% and 38% of girls and boys, respectively, were short for age, i.e., fell below the third percentile of height for age [34]. This might indicate that the low vitamin D levels in our study population were due to malnutrition. While vitamin D deficiency has been associated with overweight or obesity [15], body weights below the third percentile for height, as observed for one girl and two boys, cannot exclude poor nourishment. Ninety and 70% of the children had below recommended levels for vitamin D and A, respectively, despite living in a country with “thirteen months of sunshine” [14]. The low vitamin A levels further support our assumption that the children were malnourished. Ethnicity in Addis Ababa is heterogeneous and therefore ethnic and genetic differences, as well as environmental factors, that affect height must be considered [35].

While the short treated girls had significantly lower 25(OH)D levels than the non-treated girls, 25(OH)D levels in short treated boys were not significantly different from non-treated boys. As expected, some of the treated girls within the normal height and weight percentiles had lower 25(OH)D levels than non-treated girls. Independent of other factors, it appears that low vitamin D levels predispose girls more than boys to illness for which antimicrobial treatment is prescribed.

Individual responsiveness to vitamin D, mutations in the vitamin D receptor gene, epigenetic alterations, and sex differential mechanisms in the immune response might explain why girls are more susceptible than boys to low vitamin D levels [36,37,38]. Polymorphism of the vitamin D receptor gene is associated with serum 25(OH)D levels [39] and the receptor genotype influences the risk of upper and lower respiratory tract infections [40]. Serum levels of cathelicidin, a vitamin D-dependent antimicrobial peptide, were reported to be lower among Afro-Americans than Caucasians [41], and also lower in girls compared to boys with similar vitamin D levels [42]. The measured serum level of 25(OH)D might, however, not reflect the local concentration for the activation of vitamin D receptors and individual responsiveness [38]. The protective role of vitamin D in infection is complex and still not fully understood.

Recent studies on genetically modified mice have shown that vitamin D may protect female but not male mice against experimental autoimmune encephalomyelitis [43]. Several studies in humans indicate that micronutrients, when given to pregnant women, may be more beneficial to female than male offspring [44], suggesting sex differential activation of vitamin D responsive genes. In addition, vitamin A supplements given to young children when vaccinated against measles have sex differential immunomodulatory effects [45]. Boys are in general more susceptible to many infectious pathogens, while girls have an overall higher innate inflammatory response and Th1 profile [36,46]. Vitamin D can suppress cytokine levels and induce the activation of antimicrobial peptides [47], and may thus have different effects on sex differential immune responses. Environmental factors, behaviour, and nutrition may all influence sex-related differences in immune responses [36,37,46].

A relationship between poor social status and susceptibility to pneumonia and malnutrition was observed in The Gambia [48]. In contrast, there was no obvious single socio-economic factor associated with how the “sick” and “well” children coped with infection, or why the “sick” children received antimicrobial treatment in our study. The prescription of antimicrobials is highly variable in our setting as there are no uniform guidelines or standardized training programs for health professionals. The tendency to prescribe is affected by several factors, including parental influence, type of service, income, and clinical presentation. The influence of socio-economic factors, exposure to sunlight, and the health effects of providing vitamin D supplements to preschool children in Ethiopia warrant further investigation.

## Figures and Tables

**Table 1:** Demographics of children included, number of children providing biological samples, and number of children receiving vaccines before study start.

	All Children	Girls	Boys
*All children at study start*	95	48	47
Age (months) ^1^	51	50	54
Weight (kg) ^1^	14.8	14.2	15.0
Height (m) ^1^	1.0	1.0	0.99
BMI (kg/m^2^) ^1^	15.1	14.8	15.2
Serum 25(OH)D (nmol/l) ^1^	35.2	35.3	35.0
Serum vitamin A (µmol/l) ^1^	0.58	0.60	0.55
Serum vitamin B12 (pmol/l) ^1^	472	430	502
*Number of children providing additional samples*			
Paired plasma samples at study start/end	70	39	31
Paired PBMC samples at study start/end	63	34	29
Plasma samples for measurement of 25(OH)D and vitamin A at study start	95	48	47
Plasma samples for measurement of vitamin B12 at study start	93	47	46
*Number of children receiving vaccines*			
BCG vaccine	88	46	42
Measles vaccine	78	39	39
DTP ^2^ vaccine × 3	81	42	39
HepB ^3^ and HiB ^4^ vaccines	77	39	38
Oral polio vaccine × 3	86	44	42
Pneumococcal vaccine 1–3 doses	3	2	1

^1^ Median, ^2^ Diphtheria, Tetanus, Pertussis, ^3^ Hepatitis B, ^4^ *Haemophilus influenzae* type B (Hib).

**Table 2:** Medical history of the 95 included children during the one-year follow-up period.

	All Children	Girls	Boys
Treated with systemic antimicrobials	50	25	25
1–2 courses of antimicrobials	31	16	15
3–6 courses of antimicrobials	19	9	10
4–6 visits Medical Doctor	16	9	7
Any airway infection	43	22	21
Pneumonia	14	7	7
Pulmonary tuberculosis	2	0	2
Intestinal parasites/amoebiasis	7	5	2
Acute gastroenteritis	9	5	4
Urinary tract infection	2	2	0
Not treated with systemic antimicrobials	45	23	22
No visit Medical Doctor	34	17	17
Any airway infection	3	1	2
Skin disorder	8	5	3
Herpes simplex	1	1	0

**Table 3:** Number of children (percent), out of 70, with plasma IgG-antibodies to specific airway pathogens at the start, and numbers with new antibodies after 12 months.

	Antibodies at Start		New Antibodies	
Pathogen	All Children	Girls/Boys	All Children	Girls/Boys
Parainfluenza virus 1, 2, 3	65 (93)	39/26	4	0/4
Adenovirus	68 (97)	37/31	2	2/0
Respiratory syncytial virus	65 (93)	37/28	4	2/2
*Legionella pneumophila*	23 (33)	14/9	12	8/4
*Mycoplasma pneumoniae*	22 (32)	11/11	10	6/4
*Coxiella burnetii*	33 (47)	16/17	5	5/0
*Chlamydophila pneumoniae*	15 (21)	9/6	10	8/2

**Table 4:** Number of children (percent) out of 95 with plasma levels below recommended limits for vitamins D and A.

Substance	Lower Limits	Children (Percent)	Girls/Boys
25(OH)D	50 nmol/L	85 (89)	44/41
Vitamin A	0.7 μmol/L	70 (74)	32/38

## Supplementary Materials

The following are available online at https://www.mdpi.com/2072-6643/11/3/575/s1, Figure S1: 25(OH)D levels related to multiple infections and antimicrobial courses.

## References

[1] Dembinski J.L., Mihret A., Yimer S.A., Tessema B., Trieu M.C., Tarekegn A., Getachew N., Cox R.J., Oftung F., Haneberg B. (2017). High Prevalence of Humoral and Cellular Immunity to Influenza Viruses in Preschool Children Living in Addis Ababa, Ethiopia. Open Forum Infect. Dis..

[2] O’Brien K.L., Walters M.I., Sellman J., Quinlisk P., Regnery H., Schwartz B., Dowell S.F. (2000). Severe pneumococcal pneumonia in previously healthy children. the role of preceding influenza infection. Clin. Infect. Dis..

[3] Robinson K.M., Kolls J.K., Alcorn J.F. (2015). The immunology of influenza virus-associated bacterial pneumonia. Curr. Opin. Immunol..

[4] Ampofo K., Bender J., Sheng X., Korgenski K., Daly J., Pavia A.T., Byington C.L. (2008). Seasonal invasive pneumococcal disease in children. role of preceding respiratory viral infection. Pediatrics.

[5] Grijalva C.G., Griffin M.R., Edwards K.M., Williams J.V., Gil A.I., Verastegui H., Hartinger S.M., Vidal J.E., Klugman K.P., Lanata C.F. (2014). The role of influenza and parainfluenza infections in nasopharyngeal pneumococcal acquisition among young children. Clin. Infect. Dis..

[6] Madhi S.A., Klugman K.P. (2004). A role for Streptococcus pneumoniae in virus-associated pneumonia. Nat. Med..

[7] World Health Organization (2018). Children: Reducing Mortality.

[8] Brooks W.A., Goswami D., Rahman M., Nahar K., Fry A.M., Balish A., Iftekharuddin N., Azim T., Xu X., Klimov A. (2010). Influenza is a major contributor to childhood pneumonia in a tropical developing country. Pediatr. Infect. Dis. J..

[9] Emukule G.O., Paget J., van der Velden K., Mott J.A. (2015). Influenza-Associated Disease Burden in Kenya. A Systematic Review of Literature. PLoS ONE.

[10] Adinew Y.M., Feleke S.A., Mengesha Z.B., Workie S.B. (2017). Childhood Mortality: Trends and Determinants in Ethiopia from 1990 to 2015—A Systematic Review. Adv. Public Health.

[11] De Oliveira L.H., Camacho L.A., Coutinho E.S., Martinez-Silveira M.S., Carvalho A.F., Ruiz-Matus C., Toscano C.M. (2016). Impact and Effectiveness of 10 and 13-Valent Pneumococcal Conjugate Vaccines on Hospitalization and Mortality in Children Aged Less than 5 Years in Latin American Countries: A Systematic Review. PLoS ONE.

[12] Olarte L., Barson W.J., Barson R.M., Romero J.R., Bradley J.S., Tan T.Q., Givner L.B., Hoffman J.A., Lin P.L., Hulten K.G. (2017). Pneumococcal Pneumonia Requiring Hospitalization in US Children in the 13-Valent Pneumococcal Conjugate Vaccine Era. Clin. Infect. Dis..

[13] Muhe L., Lulseged S., Mason K.E., Simoes E.A. (1997). Case-control study of the role of nutritional rickets in the risk of developing pneumonia in Ethiopian children. Lancet.

[14] Wakayo T., Belachew T., Vatanparast H., Whiting S.J. (2015). Vitamin D deficiency and its predictors in a country with thirteen months of sunshine: The case of school children in central Ethiopia. PLoS ONE.

[15] Wakayo T., Whiting S.J., Belachew T. (2016). Vitamin D Deficiency is Associated with Overweight and/or Obesity among Schoolchildren in Central Ethiopia: A Cross-Sectional Study. Nutrients.

[16] Madhun A.S., Akselsen P.E., Sjursen H., Pedersen G., Svindland S., Nostbakken J.K., Nilsen M., Mohn K., Jul-Larsen A., Smith I. (2010). An adjuvanted pandemic influenza H1N1 vaccine provides early and long term protection in health care workers. Vaccine.

[17] Lee V.J., Chen M.I., Yap J., Ong J., Lim W.Y., Lin R.T., Barr I., Ong J.B., Mak T.M., Goh L.G. (2011). Comparability of different methods for estimating influenza infection rates over a single epidemic wave. Am. J. Epidemiol..

[18] Kolberg J., Aase A., Bergmann S., Herstad T.K., Rodal G., Frank R., Rohde M., Hammerschmidt S. (2006). Streptococcus pneumoniae enolase is important for plasminogen binding despite low abundance of enolase protein on the bacterial cell surface. Microbiology.

[19] Savic M., Dembinski J.L., Kim Y., Tunheim G., Cox R.J., Oftung F., Peters B., Mjaaland S. (2016). Epitope specific T-cell responses against influenza A in a healthy population. Immunology.

[20] Dembinski J.L., Hungnes O., Hauge A.G., Kristoffersen A.C., Haneberg B., Mjaaland S. (2014). Hydrogen peroxide inactivation of influenza virus preserves antigenic structure and immunogenicity. J. Virol. Methods.

[21] Herrador Z., Sordo L., Gadisa E., Buno A., Gomez-Rioja R., Iturzaeta J.M., de Armas L.F., Benito A., Aseffa A., Moreno J. (2014). Micronutrient deficiencies and related factors in school-aged children in Ethiopia: A cross-sectional study in Libo Kemkem and Fogera districts, Amhara Regional State. PLoS ONE.

[22] Jovanovich A.J., Ginde A.A., Holmen J., Jablonski K., Allyn R.L., Kendrick J., Chonchol M. (2014). Vitamin D level and risk of community-acquired pneumonia and sepsis. Nutrients.

[23] Usuf E., Bottomley C., Adegbola R.A., Hall A. (2014). Pneumococcal carriage in sub-Saharan Africa--a systematic review. PLoS ONE.

[24] Trzcinski K., Thompson C.M., Srivastava A., Basset A., Malley R., Lipsitch M. (2008). Protection against nasopharyngeal colonization by Streptococcus pneumoniae is mediated by antigen-specific CD4+ T cells. Infect. Immun..

[25] Wilson R., Cohen J.M., Jose R.J., de Vogel C., Baxendale H., Brown J.S. (2015). Protection against Streptococcus pneumoniae lung infection after nasopharyngeal colonization requires both humoral and cellular immune responses. Mucosal Immunol..

[26] Moe N., Pedersen B., Nordbo S.A., Skanke L.H., Krokstad S., Smyrnaios A., Dollner H. (2016). Respiratory Virus Detection and Clinical Diagnosis in Children Attending Day Care. PLoS ONE.

[27] Muenchhoff M., Goulder P.J. (2014). Sex differences in pediatric infectious diseases. J. Infect. Dis..

[28] McNally J.D., Nama N., O’Hearn K., Sampson M., Amrein K., Iliriani K., McIntyre L., Fergusson D., Menon K. (2017). Vitamin D deficiency in critically ill children: A systematic review and meta-analysis. Crit. Care.

[29] Mamani M., Muceli N., Ghasemi Basir H.R., Vasheghani M., Poorolajal J. (2017). Association between serum concentration of 25-hydroxyvitamin D and community-acquired pneumonia: A case-control study. Int. J. Gen. Med..

[30] Li W., Cheng X., Guo L., Li H., Sun C., Cui X., Zhang Q., Song G. (2018). Association between serum 25-hydroxyvitamin D concentration and pulmonary infection in children. Medicine (Baltimore).

[31] Martineau A.R., Jolliffe D.A., Hooper R.L., Greenberg L., Aloia J.F., Bergman P., Dubnov-Raz G., Esposito S., Ganmaa D., Ginde A.A. (2017). Vitamin D supplementation to prevent acute respiratory tract infections: Systematic review and meta-analysis of individual participant data. BMJ.

[32] Urashima M., Segawa T., Okazaki M., Kurihara M., Wada Y., Ida H. (2010). Randomized trial of vitamin D supplementation to prevent seasonal influenza A in schoolchildren. Am. J. Clin. Nutr..

[33] Arihiro S., Nakashima A., Matsuoka M., Suto S., Uchiyama K., Kato T., Mitobe J., Komoike N., Itagaki M., Miyakawa Y. (2019). Randomized Trial of Vitamin D Supplementation to Prevent Seasonal Influenza and Upper Respiratory Infection in Patients with Inflammatory Bowel Disease. Inflamm. Bowel Dis..

[34] World Health Organization (2018). The WHO Child Growth Standards.

[35] Jelenkovic A., Sund R., Hur Y.-M., Yokoyama Y., Hjelmborg J.v.B., Möller S., Honda C., Magnusson P.K.E., Pedersen N.L., Ooki S. (2016). Genetic and environmental influences on height from infancy to early adulthood: An individual-based pooled analysis of 45 twin cohorts. Sci. Rep..

[36] Fish E.N. (2008). The X-files in immunity: Sex-based differences predispose immune responses. Nat. Rev. Immunol..

[37] Klein S.L., Roberts C.W. (2015). Sex and Gender Differences in Infection and Treatments for Infectious Diseases.

[38] Lang P.O., Aspinall R. (2017). Vitamin D Status and the Host Resistance to Infections: What It Is Currently (Not) Understood. Clin. Ther..

[39] Santos B.R., Mascarenhas L.P., Satler F., Boguszewski M.C., Spritzer P.M. (2012). Vitamin D deficiency in girls from South Brazil: A cross-sectional study on prevalence and association with vitamin D receptor gene variants. BMC Pediatr..

[40] Jolliffe D.A., Greiller C.L., Mein C.A., Hoti M., Bakhsoliani E., Telcian A.G., Simpson A., Barnes N.C., Curtin J.A., Custovic A. (2018). Vitamin D receptor genotype influences risk of upper respiratory infection. Br. J. Nutr..

[41] Liu P.T., Stenger S., Li H., Wenzel L., Tan B.H., Krutzik S.R., Ochoa M.T., Schauber J., Wu K., Meinken C. (2006). Toll-like receptor triggering of a vitamin D-triggering response. Science.

[42] Stukes T.M., Shary J.R., Wei W., Ebeling M.D., Dezsi K.B., Shary F.S., Forestieri N.E., Hollis B.W., Wagner C.L. (2016). Circulating Cathelicidin Concentrations in a Cohort of Healthy Children: Influence of Age, Body Composition, Gender and Vitamin D Status. PLoS ONE.

[43] Krementsov D.N., Asarian L., Fang Q., McGill M.M., Teuscher C. (2018). Sex-Specific Gene-by-Vitamin D Interactions Regulate Susceptibility to Central Nervous System Autoimmunity. Front. Immunol..

[44] Klein S.L., Flanagan K.L. (2016). Sex differences in immune responses. Nat. Rev. Immunol..

[45] Jensen K.J., Fisker A.B., Andersen A., Sartono E., Yazdanbakhsh M., Aaby P., Erikstrup C., Benn C.S. (2016). The effects of vitamin A supplementation with measles vaccine on leucocyte counts and in vitro cytokine production. Br. J. Nutr..

[46] Vom Steeg L.G., Klein S.L. (2016). SeXX Matters in Infectious Disease Pathogenesis. PLoS Pathog..

[47] Esposito S., Lelii M. (2015). Vitamin D and respiratory tract infections in childhood. BMC Infect. Dis..

[48] Howie S.R., Morris G.A., Tokarz R., Ebruke B.E., Machuka E.M., Ideh R.C., Chimah O., Secka O., Townend J., Dione M. (2014). Etiology of severe childhood pneumonia in the Gambia, West Africa, determined by conventional and molecular microbiological analyses of lung and pleural aspirate samples. Clin. Infect. Dis..

