# Supplementary material for: Vitamin D Deficiency is Associated with Increased Use of Antimicrobials among Preschool Girls in Ethiopia
Source: Nutrients. 2019 Mar 7;11(3):575. doi: 10.3390/nu11030575 (PMC6471093; doi:10.3390/nu11030575)
Supplement: Supplementary file 1 [file nutrients-11-00575-s001.pdf]

Supplementary materials

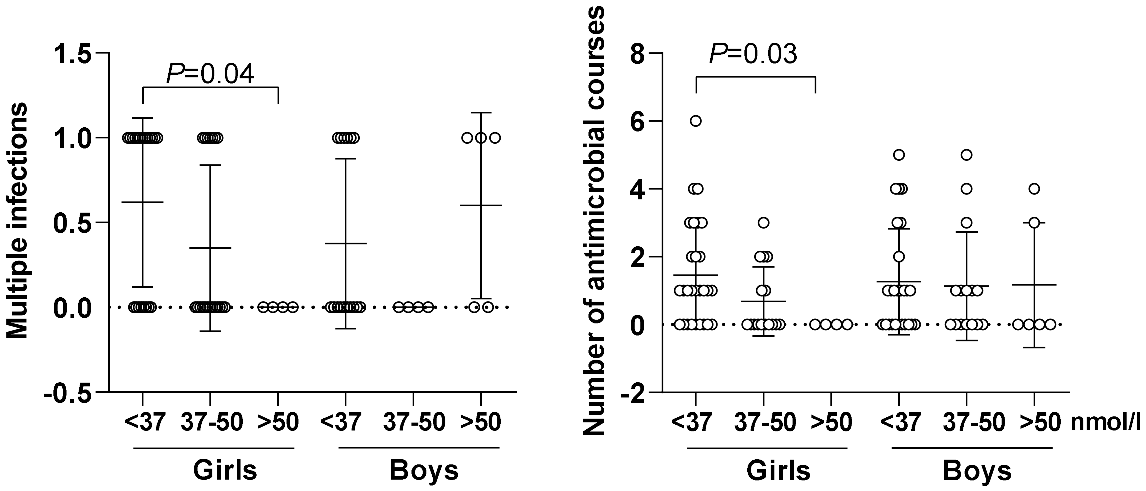

S Figure 1. 25(OH)D levels related to multiple infections and antimicrobial courses. (A) The relation between serum 25(OH)D levels and multiple infections. Multiple infections were defined as more than one infection (score 1). Girls and boys without or only one infection were categorized as 0. (B) The relation between serum 25(OH)D levels and number of antimicrobial treatment courses during the study period.
